# Vertical InGaN Light-Emitting Diode with Hybrid Distributed Bragg Reflectors

**DOI:** 10.1021/acsomega.3c10055

**Published:** 2024-07-02

**Authors:** Guo-Yi Shiu, Ying Ke, Kuei-Ting Chen, Cheng-Jie Wang, Yu-Cheng Kao, Hsiang Chen, Jung Han, Chia-Feng Lin

**Affiliations:** † Department of Materials Science and Engineering, National Chung Hsing University, Taichung 402202, Taiwan; ‡ Department of Applied Materials and Optoelectronic Engineering, National Chi Nan University, Puli 545301, Taiwan; § Department of Electrical Engineering, Yale University, 15 Prospect St, New Haven, Connecticut 06511, United States

## Abstract

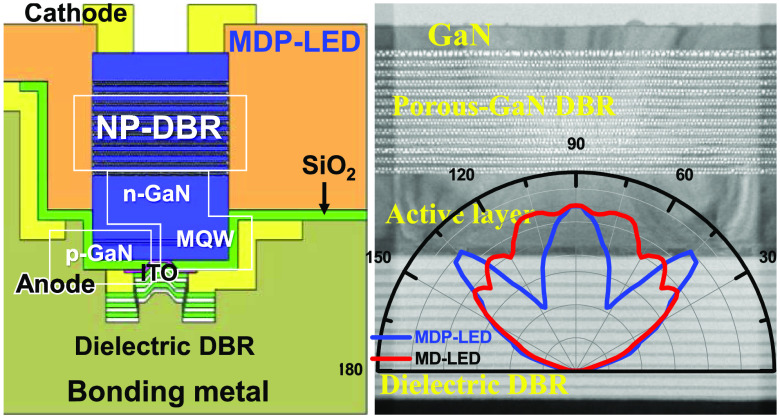

A vertical-type InGaN light-emitting diode with a resonant cavity was demonstrated with a 9 μm aperture size and a short cavity formed by hybrid distributed Bragg reflectors (DBRs). The approach involved designing epitaxial structures and utilizing an electrochemical etching process to convert heavily doped n-type gallium nitride (n^+^-GaN) layers into porous GaN layers as a porous-GaN DBR structure. Thirteen pairs of the conductive porous-GaN:Si/GaN:Si DBR structure provided a vertical current path in a vertical-type light-emitting diodes (LED) structure. The LED epitaxial layers were separated from sapphire for membrane-type LED structures through a laser lift-off process. During the free-standing membrane fabrication process, the dielectric DBR deposited on ITO/p-GaN:Mg layers was inverted from top to bottom, thereby establishing the concept of higher reflectivity for the bottom DBR compared to the porous-GaN DBR. The physical cavity length was reduced from about 2.3 μm for the LED membrane to 0.74 μm for the membrane-type LED with the embedded porous-GaN DBR structure. The divergent angles and line width of EL emission light were reduced from 124°/31.7 nm to 44°/3.3 nm due to the resonant cavity effect. The membrane-type LED structures with hybrid DBRs consisted of small divergent angles, narrow line width, and vertical current injection properties that have potential for directional emission light sources and vertical-cavity surface-emitting diode laser applications.

## 1 Introduction

Gallium nitride-based optoelectronic devices were extensively used as light-emitting diodes (LEDs),^1^ resonant-cavity light-emitting diodes (RC-LEDs),^2−4^ and vertical-cavity surface-emitting lasers (VCSELs).^5^ Face-up type structures,^6^ flip chip-type structures,^7,8^ and vertical-type structures^9^ have been reported for different current injection processes. The vertical-type LED structures were fabricated through the laser lift-off (LLO)^10,11^ process to separate the GaN epitaxial layer from sapphire substrates. This method of fabricating a top and bottom dielectric distributed Bragg reflector (DBR) was used for VCSEL structures. The embedded DBR structures were explored including epitaxial AlGaN/AlN DBR,^12^ epitaxial AlInN/GaN DBR,^13^ air gap/GaN DBR,^14^ and porous-GaN/GaN DBR.^15,16^ However, the significant lattice mismatch and minimal difference in refractive indices in AlGaN-based DBRs made their manufacturing challenging. In the case of the air gap/GaN DBR, despite a significant difference in the refractive indices of air and GaN, the lack of mechanical strength in the air gap/GaN DBR presented challenges for optical device applications. Several recent reports have revealed that the porous-GaN/GaN DBR structures were fabricated using electrochemical wet etching processes^17,18^ and the vertical etching method.^19^ A large index contrast, electrical conductivity, a significant refractive index difference, and reasonable mechanical strength were observed in porous DBR structures. In our previous report, we utilized porous GaN materials to design embedded DBR structures,^20^ aiming to achieve the fabrication of RC-LEDs and VCSELs. However, these structures primarily relied on lateral current injection, resulting in efficiency comparable to conventional face-up emitting structures. The RC-LEDs with dielectric DBRs on both top and bottom sides had been reported about using such as epitaxy in GaN substrate,^21^ GaN on silicon,^22^ and laser lift-off technology^23^ to expose the n-face GaN for depositing top and bottom DBR and realizing the vertical current path. Nevertheless, the efforts of fine-tuning the specific cavity thickness using chemical–mechanical polishing (CMP) posed challenges, hindering mass production capabilities. The GaN-based thin films without substrates were reported by using methods such as electrochemical (EC)^24−27^ wet etching, postannealing in NH_3_ processes,^28^ removing silicon from GaN/Si epitaxy,^29,30^ and utilizing two-dimensional (2D) h-BN in thermomechanical self-lift-off processes.^31^ The resulting membrane structures can be either suspended or free-standing, providing opportunities for attachment to a broader range of substrates. These thin-film structures offer benefits, including improved heat dissipation capacity and reduced internal strain.

In this paper, membrane-type InGaN structures with porous-GaN distributed Bragg reflectors (DBRs) and TiO_2_/SiO_2_ dielectric DBRs were fabricated. A laser lift-off process (LLO) was employed to separate the InGaN LED epitaxial layers from the sapphire substrate and transfer them to a target substrate. An electrochemical (EC) etching process was utilized to transform the n^+^-GaN:Si layers into porous-GaN:Si layers. The electrical conductivity of the porous-GaN:Si/n-GaN:Si DBR structure could be demonstrated to inject current vertically through the entire device. Furthermore, this structure features an accurately controlled submicron (less than 1 μm) physical cavity thickness achieved through epitaxial growth and the EC etching process. The optical properties of the membrane-type InGaN LED structure with a single dielectric DBR and double dielectric/porous-GaN hybrid DBRs were analyzed in detail.

## 2 Experimental Details

**Figure 1:**
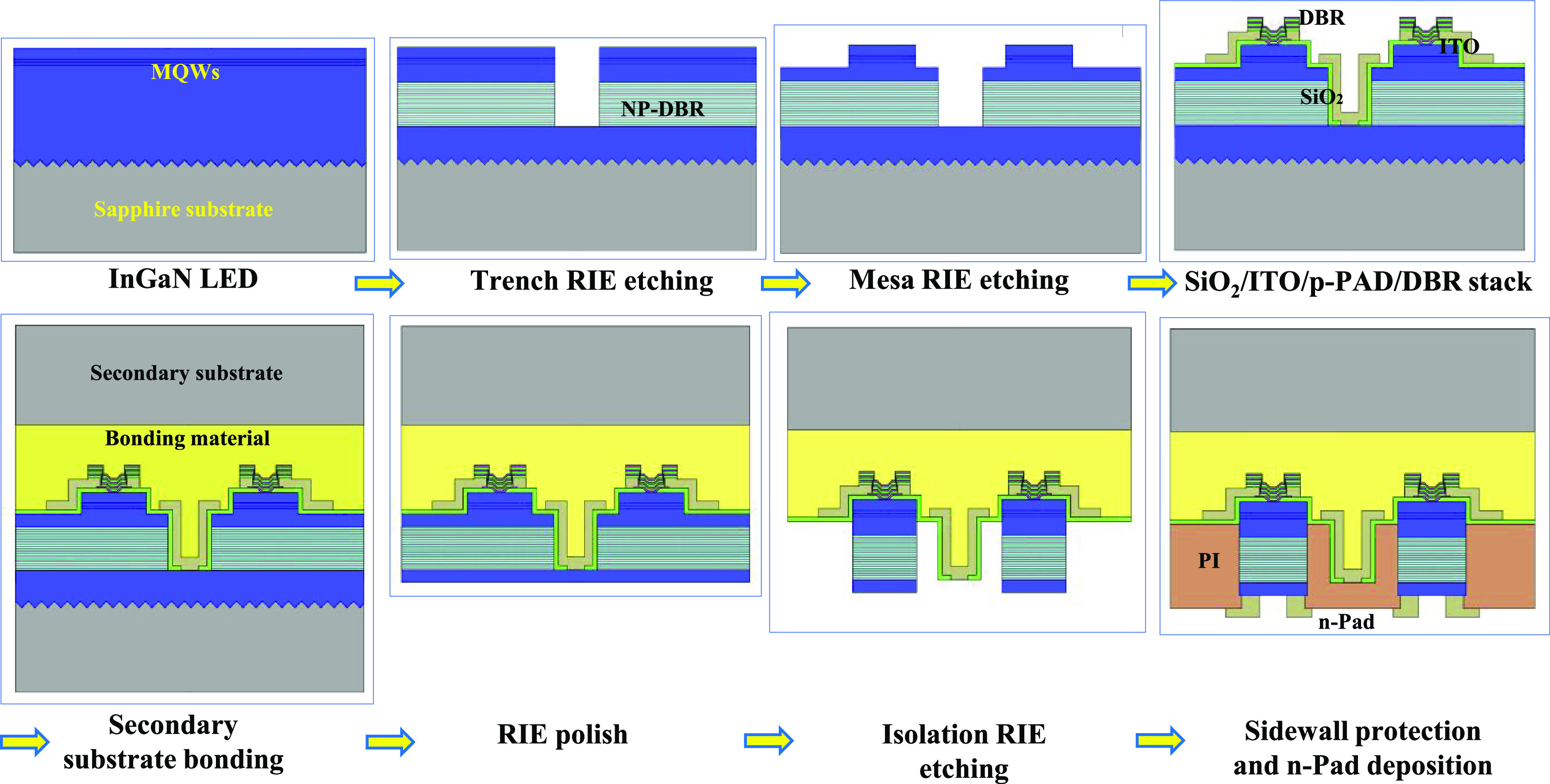
Schematic of the process flow of the MDP-LED.

The blue InGaN LED structures were grown using a metal–organic chemical vapor deposition (MOCVD) reactor. Trimethylgallium, trimethylindium, and ammonia were used as Ga, In, and N sources in the MOCVD system, respectively. The Si from silane and magnesium from bis(cyclopentadienyl)magnesium (Cp_2_Mg) served as the n- and p-type doping sources, respectively. The epitaxial growth began with a GaN nucleation layer on the c-plane (0001) sapphire substrate, followed by several layers, including an unintentionally doped GaN (u-GaN, 1 × 10^18^ cm^–3^) buffer layer, a Si-doped n-GaN (n-GaN:Si, 2 × 10^18^ cm^–3^) layer, 13 pairs of an n^+^-GaN:Si (2.5 × 10^19^ cm^–3^, 68 nm)/n-GaN:Si (2 × 10^18^ cm^–3^, 33 nm) stack structure, and LED active layers. The active layers consisted of an n-GaN:Si layer (510 nm), 10 pairs of an InGaN/GaN (3 nm/12 nm) multiple quantum wells (MQWs) structure, and a p-GaN:Mg layer (50 nm). The photolithography process and Cl_2_-based inductively coupled plasma reaction ion etching (ICP-RIE) were employed to expose the sidewalls of n^+^-GaN:Si/n-GaN:Si multilayers and to define the backside contact region. The Si-heavily doped n^+^-GaN:Si layers were transformed into conductive porous-GaN:Si layers through electrochemical (EC)^32^ wet etching in HNO_3_ solution (2.2 mol/L) at a positive bias voltage of 8 V for 4 min. Following that, the photolithography process and Cl_2_-based ICP-RIE were utilized to define the mesa area. A 100 nm thick SiO_2_ film was deposited on the defined mesa by plasma-enhanced chemical vapor deposition (PECVD), and an aperture with a diameter of 9 μm was fabricated to create a current confinement structure. Following that, a 30 nm thick indium tin oxide (ITO) layer was deposited by the sputtering process and annealed at 600 °C 3 min in an oxygen ambient to achieve transparency, conductivity, and ohmic contact properties through a rapid temperature annealing (RTA) process. Following the RTA process, metal layers were deposited on a lithographic photoresist mask with 5 nm of Cr, 70 nm of Pt, and 2000 nm of Au by electron-beam evaporation. Then, a lift-off process removed the lithographic photoresist mask and formed a patterned metal contact to extend the ITO current spreading layer. Then, 12 pairs of the TiO_2_ (40 nm)/SiO_2_ (77 nm) stack structure as a dielectric DBR structure were deposited by ion-beam-assisted deposition to form a high-reflectivity mirror. Bonding metal layers, with Cr/Pt/AuIn/Pt/Cr (2.5/50/3000/50/2.5 nm) stack structure, adherent to a receiver sapphire substrate were attached on top of the samples by using bonding technology. The laser lift-off process (266 nm pulse laser) was utilized to remove the sapphire substrate by decomposing the GaN buffer layer by focusing the laser beam on the interface between the polished sapphire substrate and the GaN buffer layer. The exposed u-GaN layer was etched by the ICP-RIE process with Cl_2_/BCl_3_ mixture sources. The photolithography process and Cl_2_-based ICP-RIE were used to isolate the porous-GaN DBR structure and the whole GaN LED structure. Then, a 3 μm thick polyimide layer was spin-coated as a sidewall insulation layer. The Cr/Pt/Au (2.5:50:2000 nm) metal layers were deposited as the cathode contact through e-beam evaporation. The lift-off LED epitaxial layers are defined as a membrane-type LED (M-LED), a membrane-type LED with a dielectric reflector on an ITO/p-GaN:Mg layer (MD-LED), and a membrane-type LED with a dielectric reflector and an embedded porous-GaN reflector (MDP-LED), respectively. The lift-off chip size was 360 μm × 360 μm with four apertures, which were fabricated on a 4” InGaN-based LED wafer. The schematic of the process flow of the MDP-LED structure is shown in Figure 1.

Surface topography and microscopic images were observed by an optical profilometer (Zeta-20) and a transmission electron microscope (TEM, JEM-2010, JEOL). The porosity of the porous-GaN DBR structure was analyzed using ImageJ open-source software. The reflectance spectra of the samples were analyzed via a fiber-optic spectrometer (USB4000, Ocean Optics). The finished devices were driven by a source measure unit (Keithley 236). Electroluminescence spectra were analyzed through an imaging spectrometer (iHR550, HORIBA) and resolved using a 300 lines/mm grating and a thermoelectrically cooled CCD detector. Far-field radiation patterns of the LEDs were measured on a rotation stage system equipped with a motor controller, scanning from 0 to 180° with a 2° step in a normal direction.

## 3 Results and Discussion

**Figure 2:**
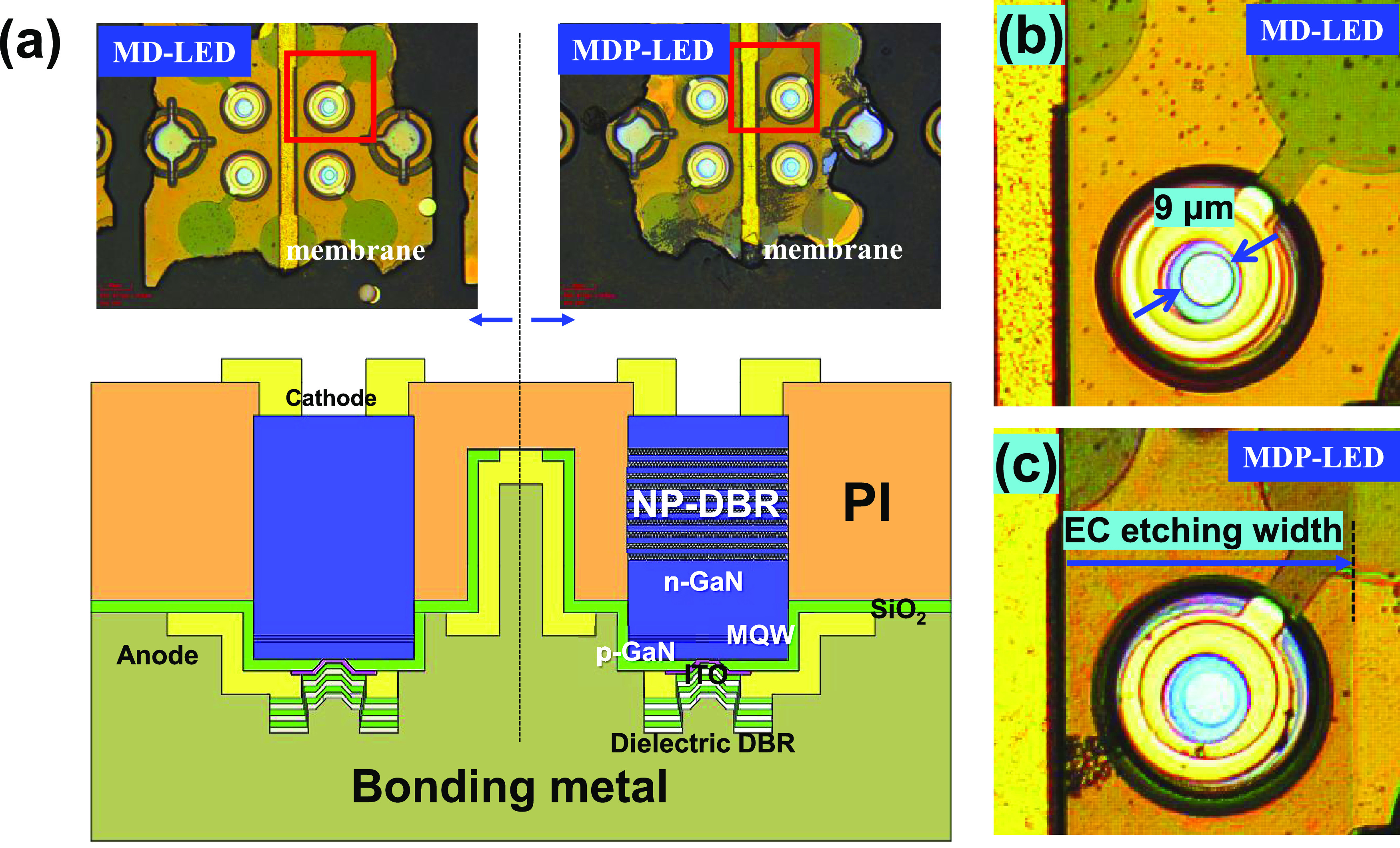
(a) Schematic and OM images of MD-LED and MDP-LED structures. The OM images of (b) the MD-LED and (c) the MDP-LED structures were observed with a 9 μm diameter aperture size.

In Figure 2a, schematic and microscopy images of a membrane-type MD-LED with dielectric DBR and MDP-LED structures with porous-GaN/dielectric hybrid DBRs were demonstrated. Both MD- and MDP-LEDs were vertical-type LED structures for the current injection. In the vertical-type MDP-LED structure, the current flows through the top N-type cathode, n-GaN:Si, conductive porous-GaN DBR, n-GaN layer, InGaN MQW active layers, p-GaN:Mg, ITO layer, bottom anode electrode, and bonding metal layer. In Figure 2b, the current injection aperture size was measured at a diameter of 9 μm in the MD-LED structure. In Figure 2c, the lateral EC etching width from the mesa sidewall was measured at about 70 μm to form the porous-GaN DBR structure in the MDP-LED structure.

**Figure 3:**
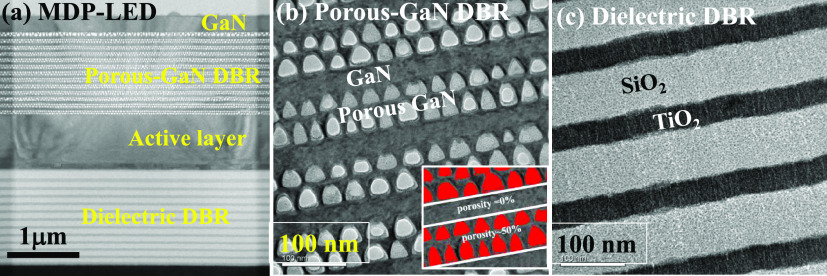
(a) TEM micrograph of the MDP-LED structure. (b). Porous-GaN and n-GaN stack structure was formed as the porous DBR structure. (c) Dielectric DBR with SiO_2_ and TiO_2_ stack structures.

In Figure 3a, the TEM micrograph of the MDP-LED structure with porous-GaN DBR and dielectric DBR structures was observed. The MDP-LED structure consisted of a 210 nm thick top n-GaN layer, a 1313 nm thick porous-GaN DBR structure, 740 nm thick short cavity layers with the n-GaN/MQW/p-GaN/ITO structure, and a 1404 nm thick TiO_2_/SiO_2_ dielectric DBR structure. In Figure 3b, the porous-GaN DBR structure consisted of a 68 nm thick porous-GaN:Si layer and a 33 nm thick n-GaN:Si layer as the stack structure. The porosity of the porous GaN layer was calculated as 50%, which indicated an effective low reflective index by mixing GaN and air. The porosity of the porous GaN layer was calculated through the open software “ImageJ” mapping on the cross-sectional SEM micrograph, as shown in the inserted image in Figure 3b. In Figure 3c, a 40 nm thick TiO_2_ layer and a 77 nm thick SiO_2_ layer as the 12-pair stack structure were deposited on the active layer, with n-GaN:Si/InGaN MQW/p-GaN:Mg layers, for the dielectric DBR structure.

**Figure 4:**
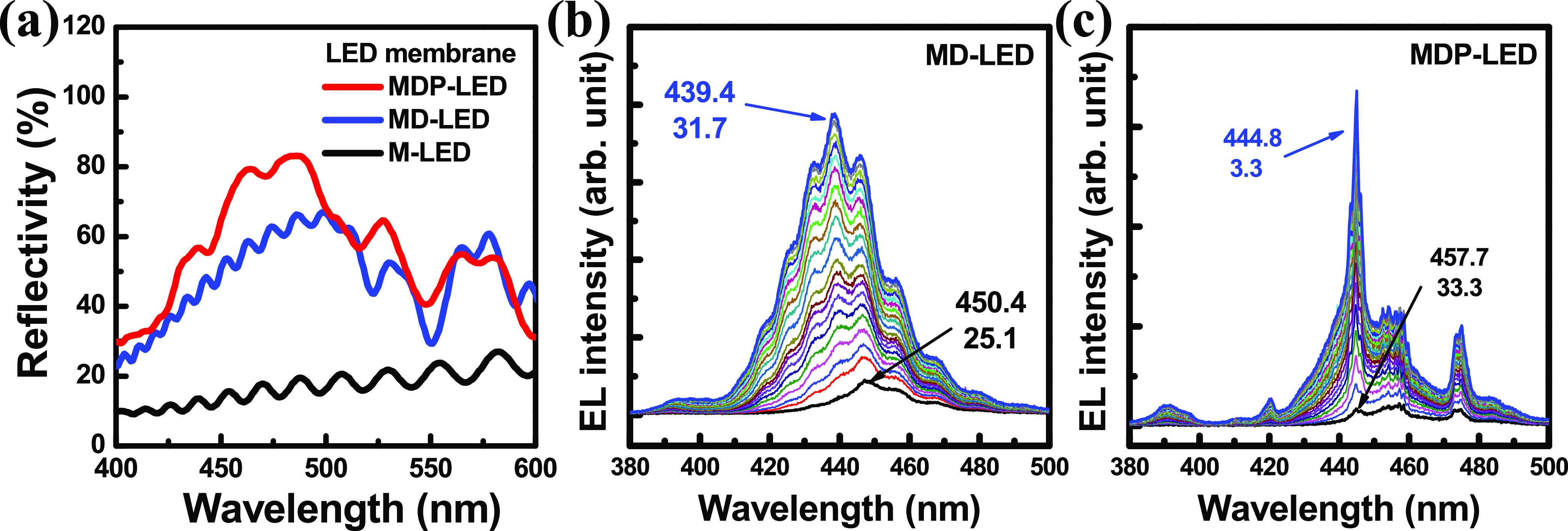
(a) Reflectance spectra were measured on the M-LED, MD-LED, and MDP-LED structures. The EL emission spectra of the (b) MD-LED and (c) MDP-LED were measured by varying the injection current.

As shown in Figure 4a, the reflectance spectra were measured on the fabricated device structures using a microreflectance measurement system. After the laser lift-off process, the free-standing epitaxial layers were separated with the sapphire substrates. The interference signal of the reflectance spectrum was observed in the M-LED structure, whose epitaxial thickness was calculated at about 2.3 μm. The thickness of the free-standing epitaxial layer was similar to the TEM result, as shown in Figure 3a. At a typical LED emission wavelength of 440 nm, the reflectivity values were measured at 13.6% for the M-LED, 44.5% for the LD-LED, and 56.7% for the MDP-LED structures. The reflectivity of the MD-LED with the dielectric DBR structure was measured at about 44.5% at 440 nm, which was higher than that of the M-LED structure (13.6%). The interference signal and the LED epitaxial layer thickness were similar in the M-LED and MD-LED structures. In the MDP-LED structure, the peak reflectivity and wavelength were measured at 484 nm and 83% on the aperture region, respectively. The density of the interference ripples was reduced in the MDP-LED compared to the MD-LED, indicating the thin cavity thickness in the MDP-LED. The physical cavity length of the MDP-LED structure was measured as 740 nm, as shown in Figure 4a. This measurement revealed a difference in cavity length between the MD-LED and the MDP-LED after forming the conductive porous-GaN:Si/n-GaN:Si DBR structure. The dielectric DBR exhibits a reflectivity of over 99% on the monitor sample through the e-gun evaporator. For the MDP-LED structure, the EL emission light from the MQW active layer was reflected by the bottom dielectric DBR and transmitted through the top porous-GaN DBR structure.

By varying the injection current from 0.1 to 2 mA, the EL emission spectra were measured for the MD-LED and the MDP-LED, as shown in Figure 4b,c, respectively. In Figure 4b, the peak wavelengths and the full width at half-maximum (fwhm) of the MD-LED had a blue shift phenomenon from 450.4/25.1 nm at 0.1 mA to 439.4/31.7 at 2 mA, respectively. The peak wavelength and the line width broaden due to the band-filling effect^33^ of increasing the injection current. The band-tilted structure in the InGaN quantum well is caused by the compressive strain-induced quantum-confined Stark effect (QCSE). By increasing the injection current into the InGaN quantum well layer, the band-filling effect in the InGaN active layer caused the EL wavelength blue shift phenomenon. The interference signal of the EL spectrum was observed in the MD-LED, in which the light was reflected between the bottom dielectric DBR and the top GaN/air interface. In the MDP-LED structure, the peak wavelengths and the fwhm of the EL emission spectra were measured at 457.7/33.3 nm at 0.1 mA injection current, as shown in Figure 4c. The wavelength peak and the fwhm of the EL spectrum were observed at 444.8 and 3.3 nm in the MDP-LED structure, respectively. The stable emission peak of the MDP-LED was observed due to the resonant cavity effect between top porous-GaN DBR and bottom dielectric DBR structures.

**Figure 5:**
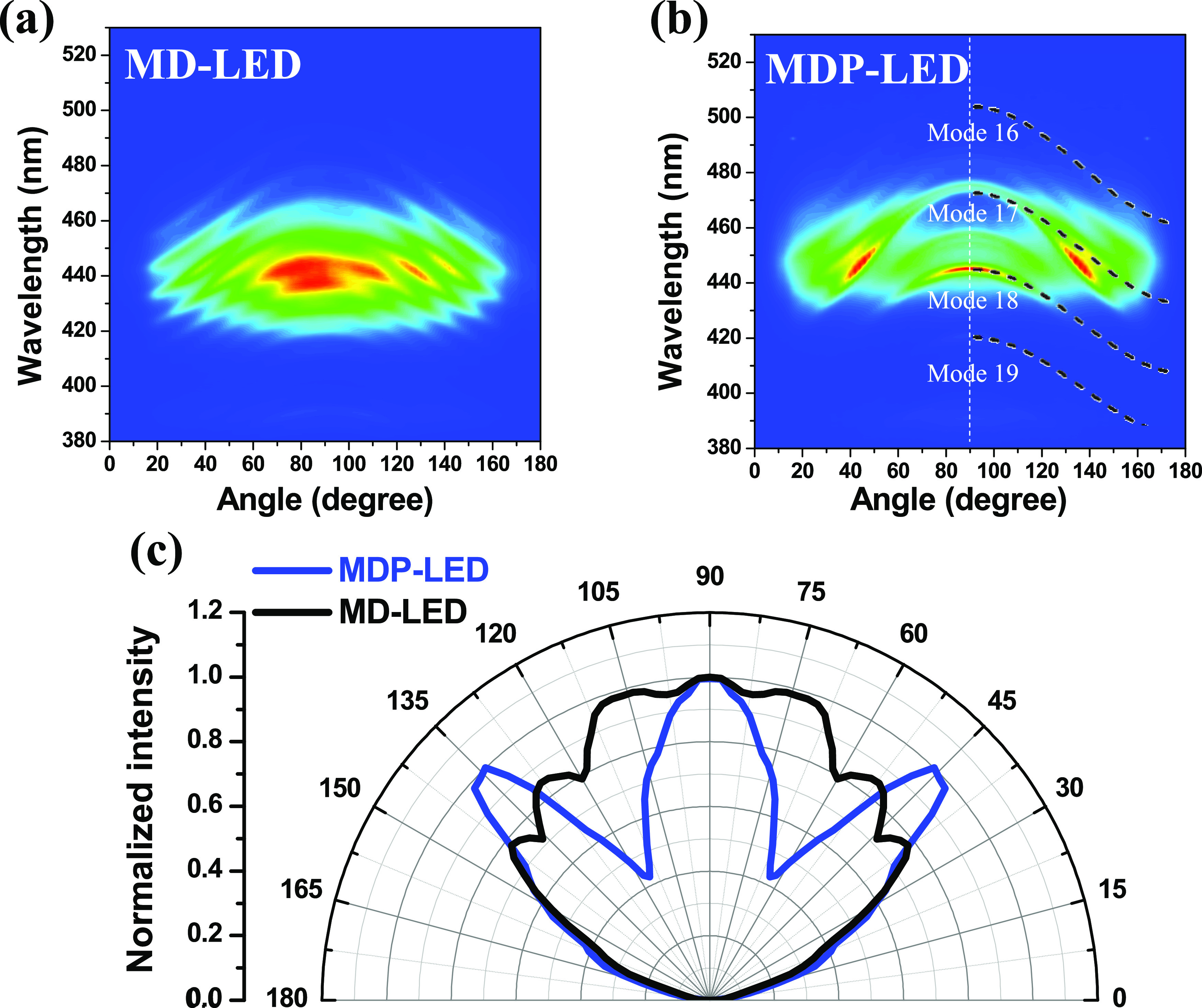
EL emission spectra as a function of the detected angles were measured on (a) MD-LED and (b) MDP-LED. (c) The far-field radiative patterns of both LED structures were measured at a 2 mA operating current.

The angle-dependent EL emission spectra of the MD-LED and MDP-LED structures are measured in Figure 5. The high-density interference line pattern was observed in the MD-LED structure, as shown in Figure 5a, where the central EL emission wavelength was located at about 440 nm. In Figure 5b, for the MDP-LED structure, the interference peak wavelengths at a 0° detected angle were observed at 420.5, 444.8, 458.6, and 475.0 nm, indicating the short optical cavity structure with hybrid DBR stack structures. This was caused by the decrease of cavity length from 2263 nm for the MD-LED to 740 nm for the MDP-LED, which was observed in the TEM micrograph in Figure 3a.

The interference formula for the Fabry–Pérot (FP) cavity is used to confirm the line patterns observed in Figure 5b. The equation is , where λ is the emission wavelength, *n* is the refractive index of GaN, *T* is the cavity thickness, *m* is the cavity mode number, and θ is the vertical tilt angle in the angle-resolved EL measurement. By using the interference formula to simulate the pattern, the optical cavity length of the MDP-LED was calculated at 1530 nm. The optical cavity length consisted of a 740 nm thick active layer and 790 nm thick for the light penetration depth in the DBR structures. The modes fitted on the graph were mode 16 to mode 19, as shown in Figure 5b. The interference line pattern of the EL far-field pattern clearly shows a significant cavity decrease from the MD-LED to MDP-LED structure. In Figure 5c, the normalized EL far-field radiative patterns of MD-LED and MDP-LED structures were shown. In the MDP-LED structure, the directional EL emission property was observed in the normal direction (90°), as shown in Figure 5c. The divergent angles of the EL intensities were reduced from 124° for the MD-LED to 44° for the MDP-LED, respectively, at a 2 mA operated current. The side peaks of the EL intensities were measured at 45 and 135°, indicating the light coupling at 17 cavity mode at the emission wavelength of the InGaN active layer. The EL emission light from the InGaN active layer was propagated between the top porous-GaN DBR and the bottom dielectric DBR structures. A strong EL peak was observed at the normal direction (90°) in the MDP-LED structure due to the resonant cavity effect in the short cavity length structure. The total light output powers of the MDP-LEDs were lower than those of the M-LEDs, which was caused by the high reflectivity on the top porous-GaN DBR structure in the light extraction direction.

## 4 Conclusions

A vertical-type InGaN-based MDP-LED has been fabricated with embedded porous-GaN DBR and dielectric DBR structures. It was separated from the sapphire substrate using the LLO process and exhibited a top porous-GaN DBR structure at the light-emitting direction. The short physical cavity length of the MDP-LED was about 740 nm, as observed from the TEM micrograph. The divergent angles of the EL intensities were reduced from 124° for the MD-LED to 44° for the MDP-LED. The membrane-type InGaN MDP-LEDs had a narrow fwhm value and a small divergent angle, having potential for flexible optoelectronic and plastic fiber-coupling light source applications.
